# Blood pressure management for stroke patients beyond the JSH 2025 guidelines

**DOI:** 10.1038/s41440-026-02765-8

**Published:** 2026-08-27

**Authors:** Kazunori Toyoda

**Affiliations:** Department of Cerebrovascular Medicine, National Cerebral and Cardiovascular Center, Suita, Japan

**Keywords:** Antihypertensive agent, Blood pressure lowering, Intracerebral hemorrhage, Ischemic stroke, Morning surge

## Abstract

As scientific evidence on blood pressure reduction in patients with chronic stroke accumulates year by year, target blood pressure levels recommended in guidelines, both in Japan and internationally, have tended to become lower. In addition, with respect to antihypertensive strategies, the Japanese Society of Hypertension Guidelines for the Management of Elevated Blood Pressure and Hypertension 2025 (JSH2025) assign a higher level of recommendation to the angiotensin receptor-neprilysin inhibitor and mineralocorticoid receptor antagonist, in addition to the conventional first-line major drugs. In patients with resistant hypertension, renal denervation can be clinically used in Japan from 2026. However, the effect of stroke prevention in these newer drugs or renal denervation remains unclear and should be further elucidated. Recommendations for blood pressure management in acute-phase stroke, particularly ischemic stroke without receiving reperfusion therapy, remain unsettled, despite the fact that the validity of relatively strict blood pressure targets has been examined in multiple clinical trials to date.

**Figure:**
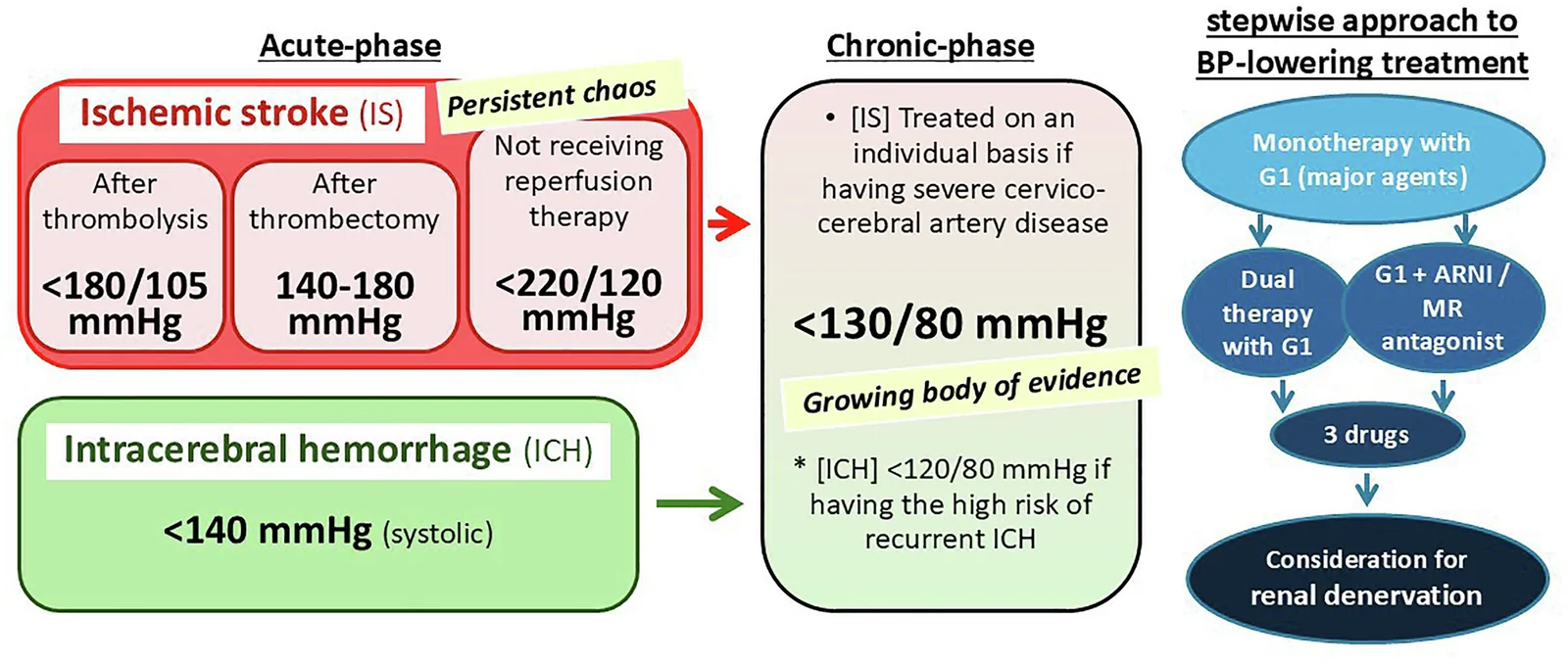
Target blood pressure for stroke patients and stepwise approach to blood pressure-lowering treatment

Target blood pressure for stroke patients and stepwise approach to blood pressure-lowering treatment

## Abstract (web summary)

Target blood pressure for stroke patients and stepwise approach to blood pressure-lowering treatment.

## Introduction

**Fig. 1:**
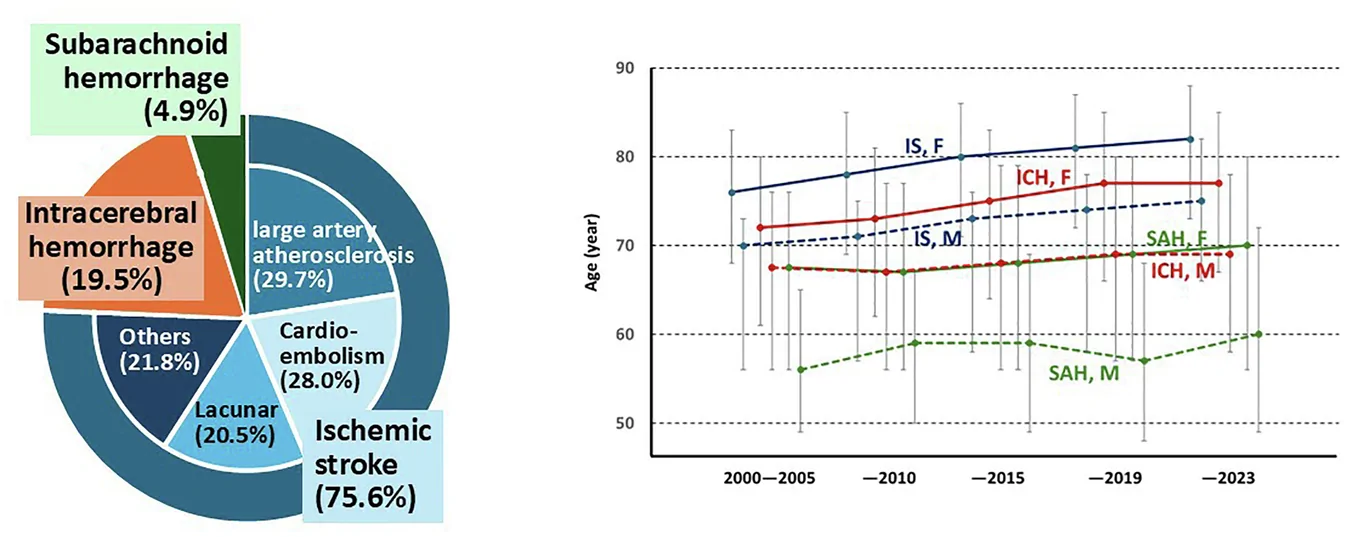
The trend in stroke features in Japan: Japan Stroke Data Bank. Left: Proportions of each stroke subtype among new-onset stroke cases from 2019 through 2023. Right: Ages (median and interquartile range) at stroke onset in each stroke subtype. Edited based on Ref [1,2]. AIS: acute ischemic stroke, ICH: intracerebral hemorrhage, SAH: subarachnoid hemorrhage

When considering blood pressure (BP) management in Japanese stroke patients, particular attention should be paid to their advanced age and the relatively high incidence of intracerebral hemorrhage (ICH). Figure 1 shows combined data from multiple publications derived from the Japan Stroke Data Bank, an ongoing, hospital-based, multicenter, prospective registry of patients with acute stroke based on a web database [1, 2]. New-onset ICH accounts for approximately 20% of all incident stroke cases, somewhat more common than in Western countries. Median ages of ischemic stroke onset, 82 years in female and 75 years in male, exceed the average life expectancy in most countries around the world.

The Japanese Society of Hypertension (JSH) updated its guidelines, the JSH Guidelines for the Management of Elevated Blood Pressure and Hypertension 2025 (JSH2025) [3], for the first time in six years, which includes a chapter on treatment of hypertension in cerebrovascular diseases as the most common hypertension-mediated organ damage. Recommendations for BP management in this guideline are basically similar with those from the United States or Europe and from the Japan Stroke Society [4–8], while partially considering the characteristics of Japanese patients. After presenting the recommendations for stroke patients in these guidelines, this review discusses, albeit prematurely, points expected to constitute major issues in the next revision.

## Blood pressure management in chronic stroke: growing body of evidence

The key updates of JSH2025 include universal target of office BP of <130/80 mmHg across populations, including chronic stroke patients [3]. To be accurate, patients with chronic ischemic stroke having severe bilateral carotid artery stenosis, occlusion of the main cerebral artery, or unassessed vessels, should be treated on an individual basis, and patients with chronic ICH with the high risk of recurrent ICH should lower BP to <120/80 mmHg.

Within the JSH guidelines which are updated every 5–6 years, the target BP for patients with chronic stroke was <140/90 mmHg until JSH 2014 guidelines [9]. In the absence of robust scientific evidence for target BP levels, a subanalysis of the Perindopril Protection Against Recurrent Stroke Study (PROGRESS) showed that the risk of stroke recurrence decreased as the mean systolic BP (SBP) during follow-up declined to around 120 mmHg [10]; based on this finding, a pragmatic threshold of approximately 140 mmHg had been adopted. JSH 2019 guidelines, where the target BP of <130/80 mmHg for chronic ischemic stroke patients was proposed for the first time, cited two papers that serve as the basis for that target value [11]. The Secondary Prevention of Small Subcortical Strokes (SPS3) trial randomly assigned lacunar stroke patients receiving aspirin alone or aspirin plus clopidogrel to two SBP-control groups with targets of 130–149 mm Hg or <130 mm Hg [12]. After follow-up for mean 3.7 years, the risk of ICH was reduced significantly by lowering SBP < 130 mm Hg, although the risk of any stroke was not. In the prospective observational Bleeding with Antithrombotic Therapy (BAT) Study in Japan, follow-up BP levels were relatively high as compared with the levels at entry in patients with intracranial hemorrhage over a median follow-up of 19 months [13]. The optimal cutoff BP level to predict impending risk of intracranial hemorrhage was ≥130/81 mm Hg using the receiver operating characteristic curve analysis. In JSH 2025, stronger evidence from the meta-analyses supporting the recommendation to lower SBP to <130 mmHg was cited. In the Recurrent Stroke Prevention Clinical Outcome (RESPECT) Study from Japan, involving 1,263 patients with a history of stroke who were randomized 1:1 to BP control to <140/90 mm Hg or to <120/80 mm Hg, the annualized rate of recurrent stroke (2.26% vs. 1.65%) was not statistically significant between the groups, whereas the rate of ICH (0.46% vs. 0.04%) was reduced in the intensive treatment group (hazard ratio 0.09, 95% confidence interval 0.01-0.70) [14]. When this finding was pooled in three previous relevant randomized controlled trials (RCTs) with stroke patients and target SBP < 125 mmHg or <130 mm Hg, including SPS3, in a meta-analysis, the risk ratio favored intensive BP control (relative risk 0.78, 95% confidence interval 0.64-0.96). We additionally updated the information from this meta-analysis and demonstrated significant reductions in recurrent stroke and major cardiovascular events in the intensive BP control arm with the target SBP < 130 mmHg or lower by integration of seven trials (Table 1) [15]. It has taken around two decades since antihypertensive therapy for patients with chronic ischemic stroke was first recommended based on the findings of the PROGRESS for the Japanese population to finally arrive at a target BP of <130/80 mmHg [16].

**Table 1:** Results of the meta-analysis based on seven randomized controlled trials comparing intensive versus standard blood pressure lowering therapy in patients with a history of stroke

Outcome	Intensive	Standard	Risk ratio (95% CI)	I^2^
Recurrent stroke	162/2555	209/2562	0.79 (0.65–0.96)	0%
Ischemic stroke	151/2234	175/2242	0.87 (0.71–1.08)	0%
Hemorrhagic stroke	8/2175	28/2191	0.33 (0.15–0.74)	0%
Major cardiovascular events	409/4020	477/4008	0.86 (0.76–0.97)	0%
All-cause death	141/2496	144/2510	0.99 (0.79–1.24)	0%
Cardiovascular death	36/1501	41/1519	0.88 (0.57–1.35)	0%
Syncope/dizziness	91/2418	71/2443	1.30 (1.00–1.68)	0%
Kidney injury	7/688	1/686	4.86 (0.84–28.27)	0%

Adapted and cited from Reference [15]

Numbers of events/total number of participants are described in the rows of Intensive and Standard

Seven randomized controlled trials studied are as follows; SPS3 (target systolic blood pressure in the intensive arm: <130 mmHg), RESPECT ( < 120 mmHg), PAST-BP ( < 130 mmHg or a 10-mmHg reduction if baseline pressure was <140 mm Hg), PRESERVE ( < 125 mmHg), PODCAST ( < 125 mmHg), a study by Park JM, et al. (<120 mmHg), and ESPRIT ( < 120 mmHg)

Most of the RCTs on BP lowering for patients with a stroke history included those with chronic ICH with lower percentage than those with chronic ischemic stroke. Thus, the target BP of <130/80 mmHg could likely be applied to ICH patients as well. In a sub-analysis of the epoch-making PROGRESS, the association of both the incidences of ischemic and hemorrhagic strokes with achieved follow-up SBP level was strong and continuous in the range of follow-up SBP from 112 to 168 mmHg; the associations were much stronger in hemorrhagic stroke [10]. While intensive BP lowering in any of SPS3, PROGRESS, and our meta-analysis suppressed the onset of ICH (or hemorrhagic stroke), it did not significantly prevent the occurrence of ischemic stroke [12, 14, 15]. As stated before, the target BP for patients with the high risk of recurrent ICH, such as those with microbleeds, receiving antithrombotics, and advanced age, is <120/80 mmHg in JSH 2025 [3].

Will target BP levels be further lowered near future? It is unlikely that general BP targets will become more stringent for the purpose of preventing ischemic stroke. The current target of <130/80 mmHg is recommended primarily based on its effect in reducing the risk of ICH, and it has not significantly reduced the risk of ischemic stroke. In the Prevention Regimen for Effectively Avoiding Second Strokes (PROFESS) trial, involving patients with recent non-cardioembolic stroke, the recurrent stroke rate was insignificantly higher in patients with follow-up SBP of 120–129 mmHg (7.2%) and significantly higher in those with the level <120 mmHg as compared to those with the level of 130–139 mmHg [17]. Such rebounding, named the J-curve phenomenon, seems to be relatively often observed in patients having large artery atherosclerosis with steno-occlusion of major cerebral arteries. We should note that ischemic stroke has diverse etiologies, the effects of BP lowering vary by subtype, and attention should be paid to cerebral hypoperfusion associated with intensive BP reduction for patients with large artery atherosclerosis. Higher percentage of syncope/dizziness after intensive BP lowing in our meta-analysis suggested cerebral hypoperfusion (Table 1) [15]. Then, in patients with lacunar infarction in whom major cerebral artery disease has been excluded, could it be possible that lowering SBP to <120 mmHg might be recommended? It might. Lacunar infarction shares vascular pathology with ICH.

How about unifying the target BP for all ICH patients to <120/80 mmHg regardless of the risk for recurrence? This may be realized simply because reduced pressure to small vessel wall seems to be protective against bleeding. We should be careful of kidney function since the Systolic Blood Pressure Intervention Trial (SPRINT) reported an impact of intensive BP lowering on acute kidney injury [18] and several studies reported an association of cerebral small vessel disease with chronic kidney disease [19, 20]. Another point of caution is that one-fifth of ICH patients were reported to have intra- or extracranial artery disease [21, 22], it means that excessive BP lowering for such ICH patients could impair cerebral circulation. The evidence underlying recommendations for the prevention of ICH is generally derived from studies that included any stroke patients, in which the proportion of ICH cases was relatively small, and there is a scarcity of studies specifically focusing on ICH patients to investigate recurrence prevention. This also makes it challenging to define the optimal BP level for ICH patients. It would be desirable to generate new evidence from Japan and Asia, where ICH is relatively more common. Although the main BP target in JSH 2025 was determined without considering for age, frailty, autonomic neuropathy (orthostatic hypotension), cognitive dysfunction, and other disorders related to both aging and stroke, they would be a barrier to appropriate BP management. Patients in such conditions are unlikely to be enrolled in RCTs; therefore, we need to identify their optimal BP levels distinct from the general targets indicated by RCTs.

## How to lower blood pressure?

**Fig. 2:**
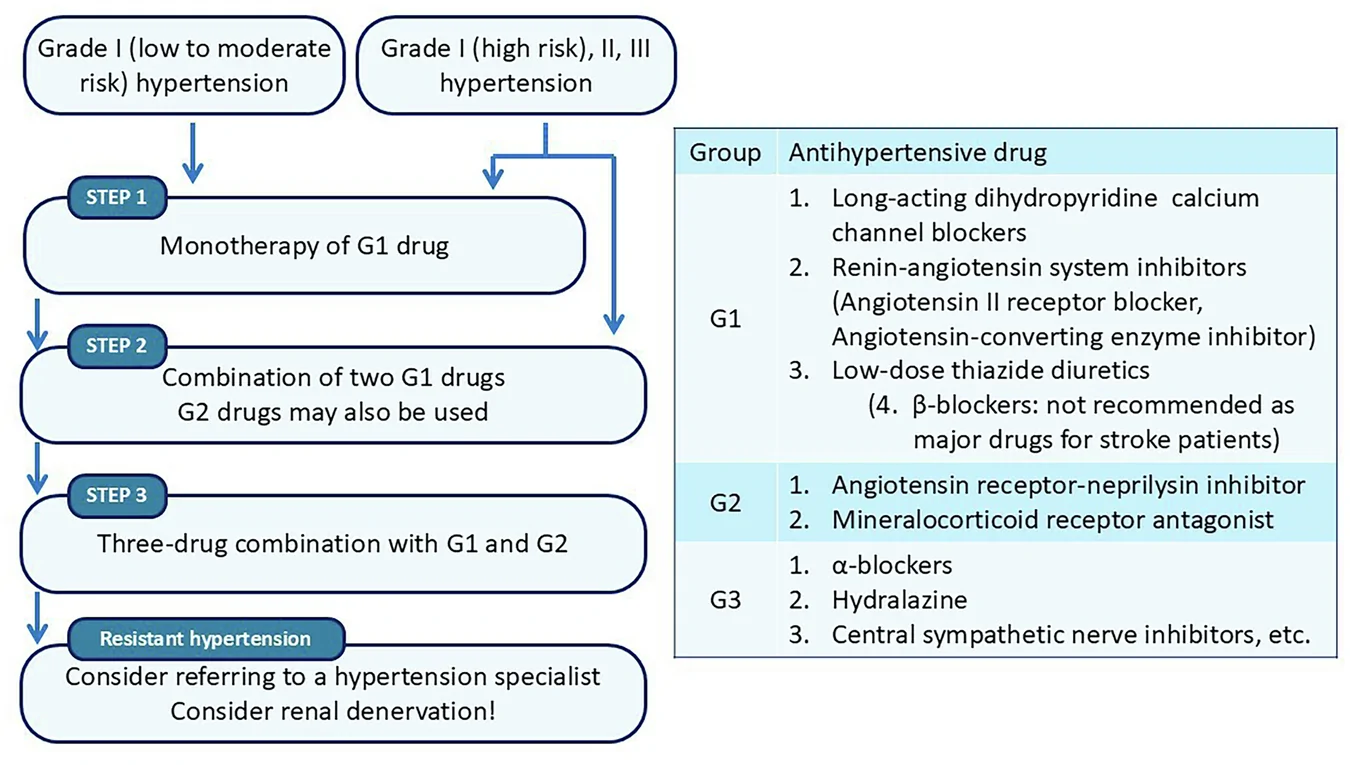
A stepwise approach to blood pressure-lowering treatment in patients with stroke. Substantially modified based on Figure 8–1 in Ref. [3]

In the JSH 2014 and 2019 guidelines, calcium channel blockers, renin-angiotensin system inhibitors (angiotensin II receptor blocker and angiotensin-converting enzyme inhibitor), and low-dose thiazide diuretics were major antihypertensive agents for stroke patients, and other drugs were not main choice [9, 11]. Note that β-blockers, another major antihypertensive agent, does not seem to be suitable for stroke patients. In JSH 2025, new second-choice drugs, angiotensin receptor-neprilysin inhibitor and mineralocorticoid receptor antagonist, are described in relatively greater detail [3]. Figure 2 shows a stepwise approach for the choice of antihypertensive agents in chronic stroke according to severity of hypertension and therapeutic resistance. This chart is largely based on that for the general hypertensive population presented in JSH 2025 [3]; however, certain parts reflect the author’s own interpretations. Monotherapy of the above-stated major agents are the first choice. If the effect is insufficient, a two-drug combination among these major agents, angiotensin receptor-neprilysin inhibitor, and mineralocorticoid receptor antagonist is chosen. The latter two novel antihypertensive agents are expected to provide a certain degree of additional BP lowering effect. An angiotensin receptor-neprilysin inhibitor is a 1:1 monocomplex of sacubitril, a prodrug of sacubitrilat, and valsartan. Neprilysin is a degrading enzyme of the natriuretic peptide system [23]. Sacubitrilat increases endogenous natriuretic peptides by inhibiting neprilysin. Thus, the antihypertensive effect of this drug is dual, the activation of endogenous natriuretic peptide function and angiotensin II receptor type 1 blockade. An angiotensin receptor-neprilysin inhibitor is used for heart failure patients in many countries but for hypertensive ones only in Japan and few other countries. Mineralocorticoid receptor antagonist drugs antagonize aldosterone-induced mineralocorticoid receptor activation in the distal kidney tubule and conjugated collecting kidney tubule, suppressing urinary potassium excretion and promoting sodium excretion, resulting in antihypertensive effects. Both angiotensin receptor-neprilysin inhibitor and mineralocorticoid receptor antagonist are promising as relatively new antihypertensive strategies. However, at present, the evidence for the antihypertensive effects of both agents remains insufficient. One reason for this is their use being largely confined to Japan, with limited global adoption. Moreover, there is a lack of robust evidence regarding stroke prevention, highlighting the need for further accumulation of solid scientific data.

The last step in Fig. 2 is ‘resistant hypertension’, uncontrolled high BP despite adequate lifestyle changes and maximal antihypertensive drug therapy (maximum tolerated dosages of three or more antihypertensive agents from different classes, including a diuretic) [24]. The prevalence of resistant hypertension ranged from 9% to 40% in adults with treated hypertension [24]. In the nationwide practice-based Japan Morning-Surge Home BP (J-HOP) study using ambulatory BP monitoring and home BP monitoring for all the participants, a hazard ratio of total cardiovascular disease risk of patients showing resistant hypertension compared to those with controlled BP using <3 classes of antihypertensive agents was 2.02 (95% confidence interval 1.38–2.94) [25]. Patients with such resistant hypertension are eligible for renal denervation, by which the afferent and efferent nerve systems of the renal artery are removed, sympathetic activation is prevented, and BP is stabilized. Randomized controlled trials comparing renal denervation with sham operation showed both effectivity in lowering BP over the entire 24-h period, including correction of the morning surge and abnormal diurnal BP variability, and safety of denervation [26]. JSH 2025 contains limited descriptions regarding renal denervation, mainly because it was not commercially approved in Japan when the guideline was published. Now, the therapy has been covered by the national health insurance system. The primary objective of BP management in the chronic phase of stroke patients is strict BP control; therefore, renal denervation may be a promising option for stroke patients with resistant hypertension in whom adequate control cannot be achieved with multiple antihypertensive agents. Scientific evidence of stroke prevention by renal denervation should be accumulated.

## Blood pressure management in acute stroke: persistent chaos

While recommendations for BP management in chronic stroke have been well established, those for acute-phase stroke, particularly ischemic stroke, remain unsettled. The BP target of ischemic stroke patients who started intravenous thrombolysis is <180/105 mmHg [3]; the target seemed to be derived from protocols for previous RCTs, not their results. The lower SBP target of 130–140 mmHg over 72 h was compared to the standard target in the Enhanced Control of Hypertension and Thrombolysis Stroke Study (ENCHANTED) and showed the safer outcome, but the functional status at 90 days was not different between the groups [27]. Similarly, the JSH 2025-recommended SBP target of patients undergoing mechanical thrombectomy is <180 mmHg [3]. In a meta-analysis published after JSH 2025 based on six RCTs comparing two stage BP levels after thrombectomy, intensive BP control to <140 mmHg or lower was associated with lower rates of good functional outcome; indicating 140-180 mmHg as an optimal level [28]. Optimal admission SBP before thrombectomy was also studied using the individual patient data from seven RCTs within the Highly Effective Reperfusion Using Multiple Endovascular Devices (HERMES) collaboration; high admission SBP was associated with worse functional outcome after stroke, but SBP did not seem to negate the effect of endovascular thrombectomy [29].

A problem is the strategy for ischemic stroke patients without reperfusion therapy (thrombolysis or thrombectomy). Guidelines in any nations uniformly recommend withholding BP lowering unless BP ≥ 220/120 mmHg (≥ 220/110 mmHg in the European Society of Cardiology guidelines) [8]. BP lowering initiated within 48–72 h after stroke onset was proven to be safe but not to improve functional outcomes in several RCTs, resulting in the recommendation of such a relatively high BP threshold [30–33]. Multiple antithrombotic medication is common in the management of acute ischemic stroke. Further studies are needed to determine whether BP targets lower than the current threshold can safely reduce antithrombotic-associated bleeding complications without increasing the risk of neurological deterioration.

The SBP target for acute ICH patients is <140 mmHg in several guidelines, including JSH2025, mainly according to the results of the Intensive Blood Pressure Reduction in Acute Cerebral Hemorrhage Trial 2 (INTERACT2) [34], although 2024 ESC guidelines set 140-160 mmHg as the target [6]. INTERACT2 was a marginally positive trial with the modest difference in the proportion of death or major disability at 90 days, corresponding to the modified Rankin Scale 3–6, between intensive (<140 mmHg, 52.0%) versus standard (<180 mmHg, 55.6%) SBP lowering. As the findings specific to Japanese or East Asians, SBP lowering to 120–160 mmHg using intravenous nicardipine appears to be well tolerated and feasible for acute ICH patients registered in the prospective observational Stroke Acute Management with Urgent Risk-factor Assessment and Improvement (SAMURAI)-ICH study in Japan [35]. In the sub-analysis of the Antihypertensive Treatment of Acute Cerebral Hemorrhage-2 (ATACH-2) trial, which Japanese hospitals participated in based on the safety results of ATACH-2, death or major disability at 90 days was 3.0% insignificantly less common in the intensive (<140 mmHg, 47.7%) than the standard (<180 mmHg, 50.7%) SBP lowering [36].

In the INTERACT4, patients with suspected stroke within 2 h after onset were randomized to prehospital SBP lowering to 130–140 mm Hg or usual BP management in the ambulance without imaging diagnosis [37]. A poor functional outcome was less common after prehospital BP lowering (common odds ratio 0.75, 95% confidence interval 0.60-0.92) in patients who were later diagnosed as having hemorrhagic stroke. The shorter the time from onset, the greater the apparent benefit of intensive BP lowering on outcomes. We should also be mindful that, in the remaining participants in this trial who were later diagnosed with ischemic stroke, a poor functional outcome was more common after prehospital BP lowering (common odds ratio 1.30, 95% confidence interval 1.06-1.60). Acute BP lowering for stroke patients in ambulance settings does not also seem to be beneficial in the Multicentre Randomised trial of Acute Stroke treatment in the Ambulance with a nitroglycerin Patch (MR ASAP) or Rapid Intervention with Glyceryl trinitrate in Hypertensive stroke Trial-2 (RIGHT-2) [38–40].

Will the target BP for acute-phase antihypertensive treatment change in the future? If intravenous thrombolysis or mechanical thrombectomy can achieve sufficient reperfusion, including at the level of the microcirculation, the risk of aggravating ischemia due to BP reduction may be reduced, and more aggressive BP lowering could lead to better outcomes. Advances in such hyperacute treatments for ischemic stroke are likely to influence BP management strategies. In ICH patients, how quickly BP lowering is initiated appears to be critical to the success of this treatment. Prehospital BP lowering is an attractive therapeutic approach; however, to gain guideline endorsement, it is crucial to be able to diagnose ICH before initiating antihypertensive treatment. Although prehospital diagnosis of ICH is already possible in some regions using CT-equipped ambulances (mobile stroke units), the development of technologies, such as blood-based biomarkers, that can accurately diagnose ICH is anticipated to allow a broader population of patients to benefit [41].

## Conclusion

BP management strategies for stroke patients recommended in JSH 2025, as well as promising antihypertensive therapies that could not be fully emphasized in these guidelines, are explained. Stroke is a condition in which hypertension plays a particularly significant role as a causative factor, and further refinements in BP-lowering strategies are needed for the prevention of stroke recurrence.
